# Advances in Neuroimmunology

**DOI:** 10.3390/brainsci7100124

**Published:** 2017-09-27

**Authors:** Donna Gruol

**Affiliations:** Neuroscience Department, The Scripps Research Institute, 10550 North Torrey Pines Road, La Jolla, CA 92037, USA; gruol@scripps.edu; Tel.: +1-858-784-7060

It is now widely accepted that an innate immune system exists within the brain and plays an important role in both physiological and pathological processes [1,2]. This neuroimmune system is comprised of brain cells that produce and secrete chemicals that are historically considered signaling factors of the peripheral immune system, such as cytokines and chemokines. Cells of the brain, primarily glia cells (e.g., astrocytes and microglia) but also neurons under some conditions, produce a large number of immune factors. In addition, endothelial cells of the brain and peripheral immune cells that enter the brain can contribute to the immune environment of the brain [3].

In general, pathological conditions are associated with elevated levels of neuroimmune factors in the brain, whereas low levels of neuroimmune factors are found in the normal brain. For example, elevated levels of neuroimmune factors in the brain have been reported for a number of conditions including brain injury, infection, neurodegenerative and psychiatric disorders, and drug abuse [4,5,6]. Considerable effort has been devoted to identifying the neuroimmune factors that play a role in these conditions, but much work is yet to be done, especially with respect to the biological actions of individual neuroimmune factors and their role in specific brain disorders.

Neuroimmune factors, like their counterpart in the periphery, produce their biological actions through interactions with cognate membrane receptor systems that translate the chemical signal through the intervention of intracellular signaling pathways. These signaling systems are complex and many have yet to be fully elucidated. Of importance is that during pathological conditions, typically multiple signaling factors are simultaneously present in the cellular environment and may activate different signaling pathways on the same cell. These intracellular pathways may interact, a complexity that is a challenge to understanding the mechanisms responsible for the biological actions associated with a particular brain condition and the development of specific therapeutic strategies. 

In this Special Issue, recent advances in an understanding of the neuroimmune system of the brain and the actions of neuroimmune factors are presented for ten areas under study; most areas are associated with pathological conditions. Together, these studies are illustrative of the breadth and status of the field, the experimental approaches being employed, and areas for future research.

The review by Gruol [7] summarizes studies on the effects of three neuroimmune factors, the proinflammatory cytokine IL-6, the chemokine CCL2, and the chemokine CXCL10, on an essential aspect of brain function: synaptic transmission. The goal of these studies is to understand the actions of specific neuroimmune factors on this process. The majority of the studies discussed employ transgenic mice that express elevated levels of a neuroimmune factor (IL-6, CCL2 or CXCL10) in the brain through increased expression by astrocytes. 

Transgenic mice that express elevated levels of IL-6 in the brain through increased astrocyte expression are also used in studies reported in the original article by Erta et al. [8]. Transgenic mice null for astrocyte IL-6 expression are also used. The goal of these studies is to identify the role of astrocyte production of IL-6 in the symptomatology of experimental autoimmune encephalomyelitis (EAE), an animal model for multiple sclerosis in humans.

The review by Mukandala et al. [9] summarizes studies that investigate the role of neuroimmune factors in acute and chronic hypoxia, and the consequences of neuroinflammation induced by hypoxia on hippocampal synaptic function. Hypoxia and neuroinflammation are two conditions that play a central role in ischemia. Complex signaling pathways involving the proinflammatory cytokine TNF-alpha and other factors are described along with their proposed roles in hypoxia and altered synaptic function associated with hypoxia.

Mori et al. [10] review the current state of knowledge on the expression and actions of two cytokines, IL-13 and IL-4, in the brain. Production of these cytokines by neurons and glia of the brain has been reported, but information is still limited. Both IL-13 and IL-4 can signal through a receptor complex comprised of IL-13 and IL-4 receptor subunits, although IL-4 also interacts with a separate IL-4 receptor. Evidence of a role for one or both of these cytokines in EAE and Parkinson’s disease is presented, along with evidence for modulatory actions on dopaminergic neurons.

Parkinson’s disease is also a topic of the review by Grimmig et al. [11]. This review focuses on the role of neuroimmunology and neuron–glia interactions in the pathophysiology of Parkinson’s disease in the context of aging. Pathological mechanisms are described along with potential therapeutic agents and strategies. Fractalkine, a protein constitutively expressed by neurons in the brain, and the antioxidant astaxanthin, a xanthophyll carotenoid that occurs naturally, are discussed as potential therapeutic agents. 

Three original articles in this Special Issue focus on the role of neuroimmune factors in the actions of drugs of abuse on the brain. Recent studies have revealed that several abused drugs, including alcohol and morphine, induce glial cells of the brain, primarily astrocytes and microglia, to secrete neuroimmune factors [2,12,13]. Microglial activation and elevated secretion of neuroimmune factors are thought to contribute to neuronal damage and cognitive dysfunction-associated excessive drug use and other pathological conditions [14]. 

The original article by Marshall et al. [15] reports results from studies on the effects of a binge pattern of alcohol exposure on microglial activation and expression of neuroimmune factors in the brain of rats. Differences in the consequences of single versus repetitive alcohol exposure on microglial activation are addressed. In the original article by Knapp et al. [16], studies are reported that examine the expression of neuroimmune mRNAs in the brain after treatment of rats to an experimental paradigm involving chronic alcohol exposure followed by alcohol withdrawal. Results from the alcohol exposure/withdrawn animals are compared to neuroimmune mRNA expression produced in rats by stress, which is a risk factor for alcohol relapse. 

Chang et al. [17] report effects of the bacterial endotoxin lipopolysaccharide (LPS) on expression of genes for proteins localized in multi-protein complexes called inflammasomes, which are important producers of neuroimmune factors and regulators of the inflammatory response. A number of different inflammasomes have been identified [18]. The studies focus on LPS-induced expression of genes for proteins housed in the inflammasomes in the context of morphine tolerance, which results from prolonged exposure to morphine. LPS is used in these studies to model invasion by a pathogen, which causes an inflammatory response. Morphine is known to affect the inflammatory response elicited by pathogens. A variety of inflammasome-related genes (e.g., for neuroimmune factors and downstream signaling partners) are examined in the brains of morphine naïve rats and rats chronically exposed to morphine in these studies. 

The review article by Liu et al. [19] focuses on another brain glial cell, the oligodendrocyte, and injury that occurs to this brain cell during HIV-1 infection. Oligodendrocytes are responsible for axonal myelination, which is essential for normal neuronal and synaptic processes that mediate brain function. Oligodendrocytes also contribute to the immunology of the brain by producing a wide range of neuroimmune mediators [20]. The process and mediators involved in oligodendrocyte and myelin damage as a consequence of HIV-1 are discussed in this article. 

Nizamutdinov and Shapiro [21] provide a comprehensive review of the traumatic brain injury (TBI), and the role of neuroimmunity and peripheral immunity in the complex pathology of this condition. Traumatic brain injury is a broad area that encompasses many types of brain injury. A number of TBI experimental models are discussed along with mechanisms of neuropathology and the involvement of neuroimmunity. Neuroimmune factors have been reported to play a critical role in TBI outcomes.
